# UTM-Chain: Blockchain-Based Secure Unmanned Traffic Management for Internet of Drones

**DOI:** 10.3390/s21093049

**Published:** 2021-04-27

**Authors:** Azza Allouch, Omar Cheikhrouhou, Anis Koubâa, Khalifa Toumi, Mohamed Khalgui, Tuan Nguyen Gia

**Affiliations:** 1 Faculty of Mathematical, Physical and Natural Sciences of Tunis, University of El Manar, Tunis 1068, Tunisia; 2 Robotics and Internet of Things Lab, Prince Sultan University, Riyadh 12435, Saudi Arabia; akoubaa@psu.edu.sa; 3 College of Computers and Information Technology, Taif University, P.O. Box 11099, Taif 21944, Saudi Arabia; o.cheikhrouhou@tu.edu.sa; 4 IRT SystemX, Paris-Saclay, 91127 Palaiseau, France; khalifa.toumi@irt-systemx.fr; 5 School of Intelligent Systems Science and Engineering, Zhuhai Campus, Jinan University, Zhuhai 519070, China; khalgui.mohamed@gmail.com; 6 National Institute of Applied Sciences and Technology, University of Carthage, Tunis 1080, Tunisia; 7 Department of Computing, University of Turku, 20500 Turku, Finland; tunggi@utu.fi

**Keywords:** Internet-of-Drones, IoD, unmanned aerial systems, UAV, unmanned traffic management, hyperledger, ground control station

## Abstract

Unmanned aerial systems (UAVs) are dramatically evolving and promoting several civil applications. However, they are still prone to many security issues that threaten public safety. Security becomes even more challenging when they are connected to the Internet as their data stream is exposed to attacks. Unmanned traffic management (UTM) represents one of the most important topics for small unmanned aerial systems for beyond-line-of-sight operations in controlled low-altitude airspace. However, without securing the flight path exchanges between drones and ground stations or control centers, serious security threats may lead to disastrous situations. For example, a predefined flight path could be easily altered to make the drone perform illegal operations. Motivated by these facts, this paper discusses the security issues for UTM’s components and addresses the security requirements for such systems. Moreover, we propose UTM-Chain, a lightweight blockchain-based security solution using hyperledger fabric for UTM of low-altitude UAVs which fits the computational and storage resources limitations of UAVs. Moreover, UTM-Chain provides secure and unalterable traffic data between the UAVs and their ground control stations. The performance of the proposed system related to transaction latency and resource utilization is analyzed by using cAdvisor. Finally, the analysis of security aspects demonstrates that the proposed UTM-Chain scheme is feasible and extensible for the secure sharing of UAV data.

## 1. Introduction

Unmanned aerial vehicles (UAVs) are an emerging technology and increasingly gaining interest for enabling several applications [1], such as package delivery [2], remote sensing [3], disaster management [4,5], search and rescue [6], traffic surveillance and management [7,8], smart cities and surveillance [9,10,11], firefighter (e.g., DJI Matrice 210 drone with Zemmuse cameras) [12], agriculture (e.g., agriculture drones including DJI AGRAS T20 for grain crop and DJI P4 multispectral for crop inspection) [13], wind turbine inspection (e.g., DJI DSLRPros Matrice 210 RTK drone with Zenmmuse or DSLRPros Matrice 300 RTK with energy and inspection package) [14], and powerline inspection (e.g., Zenith aircraft systems) [15].

As the applications of UAVs are becoming widespread, it would be necessary to monitor, control, and automate the management of their flights, to ensure a safe flight and to secure drone operation. One of the initiatives in this respect is that the National Aeronautics and Space Administration (NASA) has partnered with the US Federal Aviation Administration (FAA) to establish a UAS Traffic Management (UTM) system for the safe integration of UAVs into low-altitude airspace and managing drone traffic [16]. Furthermore, the UTM Pilot Program [17] was started in April 2017 by the FAA with the purpose to plan and execute flight paths of small UAVs. In its simple form, UTM refers to coordinating the flights of different UAVs to avoid collisions and conflicts to ensure the safe operation of multiple UAVs into a controller shared airspace. In the summer of 2019, the FAA performed UAS UTM demonstrations under the initiative of the UAS Pilot Program (UPP) for low altitude management of the airspace. Several companies, including General Electric, AirMap, and Uber, were testing solutions for the implementation of basic UTM services for UAVs. The drone’s flight paths are shared with the UTM manager in addition to other critical information about the weather and the location of the operations.

The problem becomes more challenging when drones execute their missions autonomously through the Internet. In recent years, several initiatives and research works have addressed the case of the interconnection of drones through the Internet and a concept called Internet-of-Drones [18] (IoD) has emerged. The main concept consists of controlling and monitoring the drones over the Internet and taking advantage of cloud and Internet resources to promote UAV applications. In [18], the researchers at the University of Waterloo proposed a system architecture for the Internet-of-Drones, which coordinates drones access to controlled airspace. In [19,20], the authors presented Dronemap Planner, which is a cloud-based system enabling remote control and monitoring of the drones over the Internet using Web services. Security threats were discussed, but no security solution was proposed. In [21], the authors presented an IoD platform that automates, scales drone operations, and helps to deploy intelligent drones easily. With all the aforementioned initiatives, among several others, there is an increasing trend of using drones over the Internet, in particular with the emergence of 5G networks [22]. The Internet-of-Drones paradigm provides solutions to several critical issues including, beyond-line-of-sight operations, real-time monitoring of drones, online identification, real-time localization [23], and tracking [24], unmanned traffic management [25], and user management, in addition to several other use cases. Despite these emerging applications, several challenges still remain open for research and need to be addressed. One of the most important challenges is security. In this paper, we investigate the security aspects of Unmanned Traffic Management (UTM) for UAVs.

### Problem Statement and Contribution

Although the adoption of IoD in the context of UTM provides flexibility to manage the traffic of autonomous UAVs through the Internet, this leads to many challenges such as increasing the risk of vulnerabilities that may lead to cyberattacks from different adversaries including Global Navigation Satellite System (GNSS) spoofing attacks and data manipulation. Correspondingly, serious consequences such as the increased risk of collision, incorrect advisories, and undesired or unknown flight paths can occur.

On the other hand, the UAV needs to communicate with the ground control station (GCS) to achieve an efficient path that satisfies predefined missions. However, when the communication is not secured, a third party can inject incorrect messages to falsify trajectory.

These attacks affect the confidentiality, integrity, and availability of the UTM services. Hence, it is critical to understand and address these security concerns. Ensuring that drone flight data is secure and unalterable will be essential as the industry matures, and further instructions and regulations are put in place by the government [26,27,28]. Moreover, security solutions are needed to protect the UAV flight path without affecting their performance.

In general, the flight path can be guaranteed by applying cryptographic mechanisms [29]. However, low-cost UAVs are resource-constrained in terms of computation, energy, memory, storage, and processing capacity preventing them from implementing advanced and complex cryptosecurity solutions on-board [30].

Recently, blockchain (BC) technology has evolved in cryptocurrency applications such as Bitcoin [31] and it can provide viable solutions to improve security and privacy levels thanks to its decentralized nature and traceability aspect [23,32]. Particularly, the combination of distributed systems and BC can make UAV operations safer, more secure, and flexible.

However, the limited computing capabilities of UAVs require lightweight solutions. Considering this motivation, in this paper, we propose UTM-Chain, a lightweight blockchain solution for securing the UAV flight path. The proposed solution resolves the problems previously mentioned through the following contributions:

- We carried out a survey of the security issues related to Unmanned Traffic Management (UTM).
- We leveraged the blockchain technology to secure the flight/mission plan and to guarantee safe and efficient flight planning.
- We proposed to use decentralized databases to mitigate centralized storage vulnerabilities.
- We implemented the proposed architecture using the Hyperledger Fabric [33] as a framework.
- We evaluated the performance of the proposed architecture using cAdvisor.

The rest of the paper is organized as follows. Section 2 presents some related works. Section 3 discusses an overview of the UTM system and its security requirements. In Section 4, we present the proposed solution and system architecture. In Section 5, the implementation is discussed. The performance evaluation results are described in Section 6 and Section 7 provides security analysis. Finally, we conclude and give some future works in Section 8.

## 2. Related Work

Securing the UAV flight path is a key issue to be resolved when managing the UAV traffic through the internet. Many different methods have been introduced in the literature to deal with this issue. The state-of-the-art solutions for drone flight path security are presented in Table 1.

In [34], special attention has been paid to the eavesdropping attack, and the authors tackled this problem by establishing a favorable and degraded channel for the legitimate and eavesdropping links, via a trajectory design. Similar work has also been pursued by [35], in which they suggest using a joint trajectory and transmit power design algorithm to prevent eavesdropping attacks. To improve UAV data security, the authors in [36] suggested the idea of an additional encrypted communication channel through Raspberry Pi. The channel is used to resume the UAV control in case there is an attack towards the drone. Nonetheless, the solution still has some issues such as increasing the Raspberry Pi’s CPU usage and a delay between the GCS and the Raspberry Pi. The discussed approach was not analyzed properly in terms of performance.

In the work of [37], it is possible to prevent the issue of waypoints alteration and path modifications if secure trajectories with efficient MAC protocol are applied.

In general, waypoint security can be guaranteed if the cryptographic mechanisms are applied. There has been extensive interest in this direction. In [38], RSA and ECC algorithms were used to encrypt Aerial Robotics Communication. However, its main drawback is that the public key cryptography depends on the third-party authority working with the certificates.

The work in [39] designed a lightweight IBE scheme for IoD that is resource-constrained in terms of computation and memory storage. The proposed scheme guarantees that the data shared between drones is secured.

Recently, few works were interested in using blockchain as a key technology for IoD security. The author in [40] introduced blockchain in the field of robotics to solve security issues in swarm robotics. However, only the description of the approach is quickly provided, and no implementations or simulations were realized.

In [41], the communication of autonomous unmanned air vehicles (UAV) including data exchanging can be secured by using blockchain. The work in [42] presents the ideology of exploiting the features of the blockchain to secure utilization of drones, especially in ultradense environments.

Liang et al. [43] combine public blockchain and cloud storage in their framework to guarantee the integrity of data collected using drones. A prototype of a drone system was implemented, and performance evaluation results are acceptable.

Authors in [44] introduced a blockchain-based system for proffering security and privacy to the IoD network. In [45], the authors utilized blockchain principles to detect compromised UAVs and incorrect information when a UAV is hijacked.

**Table 1:** Summary of related works.

Scheme and Year	Contribution	Technology Used	Network Model	Validation Tool	1	2	3	Limitations
Yoon et al. [36] (2017)	Enhancing UAV data security by developing an additional encrypted communication channel	AES encryption	Multi-UAV, ground station and middleware	Raspberry Pi, Aircrack-ng	N	Y	N	High overhead because of utilization of encryption channels Requiring extra hardwareHigh delay
Kapitonov et al. [41] (2017)	Developed a secure communication protocol among UAVs	Ethereum, private and public keys	Communication system between agents (DAOs) in a P2P network	Solidity, Smart contract ROS, Inter-Planetary File System; utilization of docker virtualization to implement AIRA protocol	Y	Y	Y	Issues of AIRA protocol were not discussedthere is no investigation of data privacy
Sharma et al. [42] (2017)	Securely relay drone information via blockchain	Public and private keys, UAVs, BC	UAVNET	Smart contracts	Y	N	Y	Cannot be applied to IoD
Liang et al. [43] (2017)	Ensure the secure communication and integrity of data collected from drones using blockchain and cloud storage	Chainpoint, Ubuntu, Apache	The network is composed of drone, control system, blockchain network, cloud database, and cloud server	POW, bitcoin	Y	Y	Y	It is not practical to minimize the drones’ burden by using the traditional cloud server together with blockchain technologyTheoretical Explanation
Han et al. [38] (2017)	Encrypt drone-GCS communication	Elliptic Curve Crypto-graphy (ECC) and RSA	The network is composed of one UAV and a ground station	N/A	N	N	N	Public key cryptography depends on the third-party authority
Lin et al. [39] (2018)	Propose mutual authentication between drones and ground stations for secure communication in IoD and secure data sharing among collaborating drones	Identity-based encryption (IBE)	IoD, drone, Certificate authority (CA), cloud	N/A	Y	N	N	No detailed constructions are proposed
Ferrer et al. [40] (2018)	Provide BC solution to entity validation and data confidentiality in swarm robotics	Key and digital signature crypto-graphy, UAV, BC	Drones	N/A	N	N	Y	Description of the approach is quickly provided, and no implementations or simulations were realized
Cui et al. [35] and Zhang et al. [34] (2018–2019)	Control the UAV trajectory jointly with the transmit power allocation to maximize the UAV’s average secrecy rate over a given flight time	Adjusting UAV trajectory and transmitting power by using a physical-layer based approach	A simple network of one UAV and a ground node	Simulation	Y	N	N	How this solution will work with multiple UAVs ?
Choudhary et al. [37] (2019)	Secure the trajectory of UAV using a security framework based on a deep neural network, which integrates an efficient MAC protocol controlled by Macaulay duration	MAC protocol, deep neural network	Several types of communication links are used in the system including UAV-to-UAV, UAV-to-device, and virtual	Simulation-based evaluations	N	N	N	Location privacy is not considered
García-Magari et al. [45] (2019)	Improving the security of the UAV network by using blockchain-based techniques	PoA, smart contract, asymmetric key encryption	Multi-UAV	The proposed model is validated via Agent based simulator (ABS-Security UAV), Hyperledger, supporting mobile ad-hoc network	N	Y	Y	Focus only on detecting whether UAV is hijacked or not

Note: 1: Architecture/Framework given; 2: Implementation done; 3: Blockchain-focused, Y: Yes, N: No; N/A: Not defined.

## 3. Security Issues in Unmanned Traffic Management (UTM)

UTM is a drone traffic management system, automated and located in the cloud with the mission of organizing the flight of drones registered with the FAA. The future UTM system will rely on an increase in interconnected systems based on novel technologies (IoD). However, security and vulnerability to attacks are some of the major challenges to the adoption of the UAV traffic management systems, especially when autonomous drones operate to provide near-real-time data to a GCS for timely decisions. In what follows, we address UTM security issues. We identify and classify security threats and challenges facing Unmanned traffic management systems, including those resulting from UTM components vulnerabilities, namely, UAV, GCS, cyber systems (cloud and the Internet), and data communication links between all the components as depicted in Table 2.

### 3.1. Attacks on UAV

The UAV is vulnerable to several kinds of attacks, which can be categorized into two groups: physical attacks and cyber attacks.

#### 3.1.1. Physical Attacks

Due to the drone characteristics such as shape and propellers, most drones are vulnerable in terms of their physics. When a physical attack caused by birds, drones, or flying items such as parachutes or hail occurs, the drone or its parts get broken easily. In addition, drones can be easily harmed by environments such as strong wind, high temperature, or complex flying environments having obstacles. These physical attacks increase the complexity to accomplish drone missions. It is challenging to deal with these attacks as many of them can occur suddenly [19].

#### 3.1.2. Cyber Attacks

- **Hijacking:** is the major researched area in UAV cybersecurity. The third-party can send unauthorized commands to control drones when drones are properly protected or their communication is not secured. Correspondingly, it can cause serious consequences such as failed missions.
- **GPS spoofing attacks:** GPS is used for pathfinding in transportation and enables a drone’s navigation. A drone that uses GPS is vulnerable to spoofing attacks due to the lack of encryption. In a GPS spoofing attack, fake GPS signals stronger than the original unencrypted GPS signals can be transmitted into the drone to mislead the UAV’s path. GPS spoofed signals are providing false altitude and longitude data to the drone, thus letting it change its trajectory. This attack could result in a loss of accurate positional information, which may even threaten the drone and the safety of nearby occupants in the UTM.
- **GPS jamming attacks:** Other attacks on drones involve GPS jamming attacks. The jammer may intend jamming of the GPS signal that navigates the path of the UAV. In this scenario, the drone will be unable to determine its location and trajectory.

Hence, the main concern for the UTM is that an attacker can use UAV-related vulnerabilities to threaten UTM by changing the drone flight plan, which can increase the probability of collisions with another aircraft or static obstacles.

### 3.2. Attacks on GCS

Attacks against ground control systems are mostly through software-related threats, namely, virus, malware, trojan, keylogger, etc. This class of threats could result in a loss of sensitive data or even a loss of UAV control [29].

### 3.3. Attacks on Data Communication Link

UTM entities communicate among themselves through a wireless channel. The communication link between the UAV and the ground control station is unencrypted, which makes this link highly prone to several attacks. The possible attacks on the data communication link can be listed as follows:

- **Denial of Service (DoS/ DDoS):** Denial of Service attacks compromises UTM availability, in particular by flooding the network with fake requests, thereby the network becomes interrupted, making the UTM system appear unavailable and preventing other legitimate packets from being sent. Correspondingly, the drone cannot receive authorized control messages and data, which leads to failed missions. In a DDoS attack, a large number of unauthorized packets are transmitted over the communication links by an adversary to the UAV or the GCS that can cause network congestion preventing proper communication between the UAV and the GCS.
  In attacks under denial of service categories, such as jamming, the adversary aims to disrupt the communication link between the UAV and other entities in its network through interference or collision before the reception. Particularly, the jammer within a specific radius at the frequency of the drones generates interference within radio channels. As a result, a receiver such as a ground station or a drone cannot properly receive signals transmitted by an unauthorized sender that can cause unavailable services.
- **Traffic analysis attack:** Traffic analysis attack is a passive attack, which is performed by a third party to examine the UTM traffic to get useful information from the UTM components and network. The traffic contains sensitive data exchanged between UTM nodes like mission plan, location, and telemetry data.
- **GCS Control Signals spoofing:** If the wireless communication link between the UAV and the GCS is not protected, spoof MAVLink (Micro Air Vehicle Link) commands can be sent by unauthorized parties to take over the UAV illegitimately.
- **Man in the middle:** The M-I-T-M attack can be successfully established on telemetry and Command-and-Control (C2) data links. The attacker can intercept the exchanged messages between UAV and GCS. Particularly, the exchanged packets can be captured by attackers who then can extract the important information, relaying the packet with modified data. This makes both the drone and the ground control believe that they are communicating with each other successfully without any interception.
- **Eavesdropping:** When the connection between UAVs and GCS is not secured or encrypted, an attacker can eavesdrop on the exchanged messages between these. Correspondingly, the attacker can extract information (e.g., control and command data, location of drones, and flying speed) from the exchanged messages. Eavesdropping is a passive attack; however, the extracted information via eavesdropping can be used as a foundation for active attacks such as hijacking that controls the UAVs and has a large impact on UAV missions.
- **Identity spoofing:** When the MAVLink is not encrypted, authentication credentials of the drone or GCS can be captured by the third party. Then, the third party can use the authentication credentials to send messages to the receivers.
- **False location update:** When the communication between UAVs and GCS is not secure enough, the attack can use the data link to send false UAV location data to GCS that can cause wrong trajectory and failed UAVs missions.

### 3.4. Attacks on Cyber Systems (Cloud and Internet)

Cybersystems such as the Internet and the cloud are used in the UTM system to coordinate the access and the flight of multiple UAVs. Cloud computing resources are used to offload heavy computations and support the processing and storage of streams of data originated from UAVs [46]. In general, a regular relational database such as SQL or distributed file systems can be used for storing data and information. When the database is compromised, the security of the UTM is significantly affected.

- **SQL and NoSQL injection attack:** If a database is not designed and managed properly and securely, the database may have some vulnerabilities that can be exploited by hackers who can send malicious SQL code to gain access to backend databases [47,48,49]. If the access is successfully got, they can control the webserver or manipulate the contents of the databases [50,51].
- **Insecure APIs:** In general, cloud infrastructures offer APIs for accessing and manipulating data. However, APIs may have vulnerabilities that can be exploited by attackers [50]. Particularly, software developers who design and develop APIs may use open-source code to accelerate the developing process. However, the open-sources may be insecure and have some pieces of code unknowingly tainted with cryptocurrency mining code. It is required that the API needs to support secure communication and other security algorithms to guarantee a high level of security related to authentication, access control, confidentiality/privacy, encryption, segregation of data and privileges, and error handling.
- **Malware injection:** Cross-Site Scripting is one of the most widely used ways of malware injection [49,50]. In Cross-Site Scripting, Malicious scripts including JavaScript, VBScript, ActiveX, and HTML can be injected by attackers into a vulnerable web page with the purpose of executing the malicious scripts on a web browser of a victim [52]. Correspondingly, the session cookies could be stolen or the victim could be tricked into using a malicious link.

## 4. UTM-Chain System Architecture

In this section, we present the proposed blockchain-based unmanned traffic management system.

### 4.1. Proposed System Overview

Blockchain can be described as a shared and distributed ledger of transactions [53]. Blockchain works based on the consensus mechanism that is a fault-tolerant mechanism to reach an agreement on the value of some shared data between participants. When new data is added into the blockchain, a record of added data is added to every participant’s ledger and the data cannot be edited or erased. Blockchain allows UAVs, GCSs, and users to communicate with each other securely.

The proposed UTM-Chain is a blockchain-based solution for the Internet of Drones that permits to secure UAV flight plan and store UAV’s flight data records. The features of UTM-Chain are summarized as follows:

- Decentralization: UAVs, GCS, and users can communicate and exchange data directly through a secure and trusted blockchain without any third-party entity involvement. Moreover, data is distributed across a blockchain network and stored in an off-chain database.
- Collective verification: Because of blockchain’s distributed characteristics, transaction data should be approved and verified by the participants (e.g., nodes), thus eliminating the need for a controlling authority.
- Tamper resistance: When transaction data is added into the blockchain, it is protected and cannot be tampered (e.g., modified or deleted) thanks to the cryptography. Particularly, each block of a blockchain has a cryptographic hash of the previous block. Therefore, a data of a particular block cannot be modified without the alternation of all subsequent blocks, which is almost impossible.
- Data Integrity: The data integrity ensures the accuracy and consistency of data in secure transmission and storage. In blockchain, Cryptographic Hash Functions are used to ensure the integrity of the data transmitted by IoD devices. The integrity of this data is also guaranteed by the consensus mechanism used in the block mining process [54]. On the other hand, once the data or information is written in a block, it can no longer be deleted or modified since its integrity is intrinsically provided by the way it is chained to other blocks.
- Availability: Integrating blockchain technique in IoD enables higher availability because of blockchain’s distributed characteristics, considering that multiple copies of the entire ledger are stored on each distributed node in the blockchain network. If a UAV is under attack, the rest of the blockchain nodes keep on working and data is always available.
- Data origin authentication: All nodes in blockchain are capable of verifying the authenticity of the data transmitted, with the help of digital signature cryptography. Thus, data authentication between UAVs, GCS, and users is guaranteed.
- Identity management: Identification of nodes can be achieved by using blockchain through the use of pseudonymous addresses similar to Bitcoin addresses.
- Privacy: In blockchain, transaction history is publicly available and any node can join and access details about transactions, but it cannot access identifying information about the node making those transactions [53].

The proposed UTM-Chain achieves the above objectives while adopting the following architecture.

### 4.2. The Proposed UTM-Chain Architecture Description

**Figure 1:**
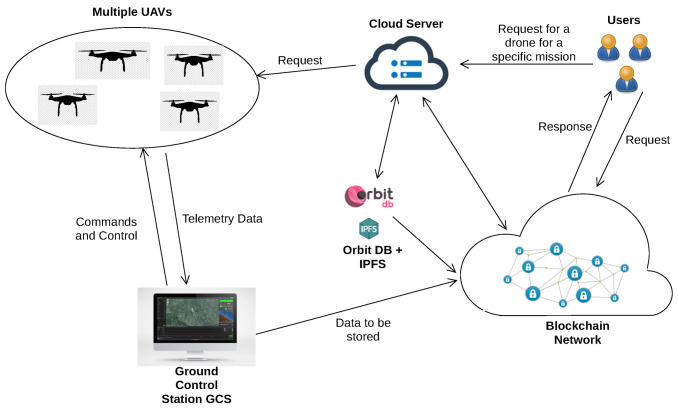
An illustration of the proposed blockchain based IoD architecture.

In this section, we present the architecture of UTM-Chain for blockchain-enabled IoD. The architecture is shown in Figure 1 and consists of five main components including multiple UAV (drones), ground control stations, users, cloud server, and the blockchain network.

The different actors of the architecture are presented in what follows:

- Multiple Unmanned Aerial Vehicles (drones): UAVs are pilotless aircraft that can operate autonomously via the on-board computer or can be remotely controlled by a pilot at the Ground Control Station (GCS). UAVs use sensors to collect different types of data such as UAVs’ speed, battery level, altitude, RGB images, thermal images. Depending on the system requirements, specific sensors can be applied. The collected data can be preprocessed or kept intact at the UAV before being sent to GCS.
- Ground Control station: GCS is responsible for receiving data from UAVs and sending out commands to control UAVs including uploading new mission commands and updating controlling parameters.
- Users: are third-parties that have access to the parameters and data of the UAVs and GCS for useful purposes.
- Blockchain Network: The main role of the blockchain network is to maintain a distributed immutable database of actions performed by users, data collected from drones, and commands from GCS. The recorded transactions are shared between nodes in the network. In our architecture, the users, UAVs, and GCSs act as nodes, storing the whole blockchain and participating in the consensus protocol to verify blocks.
- Cloud server: is responsible for offloading computation from the drone to overcome the computing resources’ limitations of the UAVs and optimize the mission execution. Correspondingly, the flying time of the drones can be extended.

We propose to use OrbitDB with the Inter-Planetary File System (IPFS) as an off-chain database. The off-chain database is used in order to store UAV data (mission information, flight status details, sensor data) that is too large to be stored in the blockchain efficiently. In fact, OrbitDB is a distributed peer-to-peer database performing with IPFS. The latter generates the hash of the data saved into OrbitDB and stores it as an immutable transaction in the blockchain.

### 4.3. Interaction Model

We design an architecture to secure the UAV flight plan and UAV data. The proposed system can be applied in different applications such as search and rescue or tracking illegal activities or abnormalities. In a search mission, a team of autonomous UAVs are deployed in an outdoor environment to search a person or object. The search mission can be defined by the system operators such as the rescue team.

**Figure 2:**
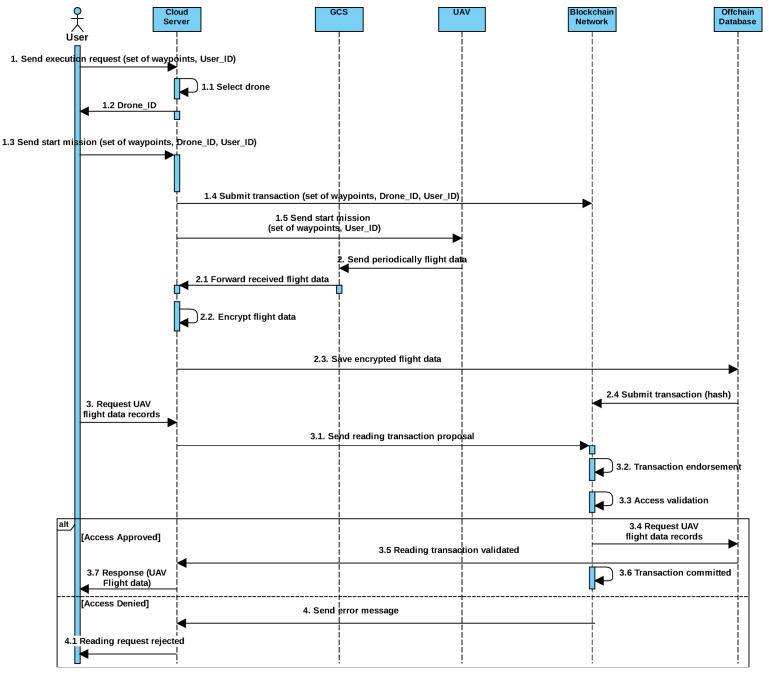
Search mission sequence diagram.

Moreover, the drone communicates and updates its information (e.g., GPS location, altitude, speed, or images) with the ground control station through the Internet. The sequence diagram of the use case related to the search mission is illustrated in Figure 2. At first, all drones are registered before they start to perform their mission.

The mission starts with the preflight phase, where one of the users defines a mission (selects locations of the target object to visit on the map) and makes an execution request to the cloud server including the location of the target object (the set of waypoints). Once the mission request is received, the cloud server will select an available UAV that is already registered to execute the mission. The set of waypoints that the drone will visit are stored in the blockchain.

Throughout the in-flight phase and during the mission, the drones constantly update their GCS about their GPS location, flight plan data such as route and destination address, mission details, and telemetry data including sensor data and images/videos. Then, the GCS forward this data to the cloud server, which, in turn, encrypts the collected data and store it in an off-chain database. The latter is a distributed database using the file-sharing protocol IPFS. When storing the data, IPFS generates the hash of each stored block.

Finally, in the post-flight phase, to be sure that the flight mission is terminated as planned, the cloud server compares the waypoints defined by the user in the preflight phase with the GPS location stored in the off-chain database in the in-flight phase. If the waypoint matches with the GPS location, the cloud informs the user about the completion of the UAV’s flight mission. Otherwise, in case of an unexpected flight termination, the user sends a request to the cloud to get historical flight data records in order to analyze data records to find the root cause of the problem. The latter sends a reading transaction proposal to the blockchain. In the blockchain network, endorsing peers run the chain code in order to validate the user access right. After validating the access, requested data is retrieved from the off-chain database. In fact, the response is sent to the cloud server and the reading transaction is committed into the blockchain. Finally, the cloud server communicates the requested UAV’s flight data to the user.

## 5. UTM-Chain Implementation

In this section, we gives a description of the tools used to implement our solution..

### 5.1. Development Environment

We use Hyperledger Fabric [55], Hyperledger Composer [56], Docker Composer, Docker Engine, and BNC [57] as a command-line tool to implement the UTM-Chain architecture. The proposed system’s configuration is depicted in Table 3. The blockchain framework is running on an Ubuntu Linux 14.04 TLS on Intel Core i-7-8500 @2.9 GHz processor with 5.4-gigabyte memory with the Hyperledger Composer, composer-rest-server, and node. In addition, docker engine is used to configure the docker image and container. Docker composer is used to configure the composer-rest-server. Moreover, we use Hyperledger Fabric, an open-source platform, to implement blockchain applications and composer-playground to create and built the business network definition. To expose the web services, composer-rest-server is used to create the REST API for the entities in the blockchain platform.

A simulated drone executed with the Ardupilot Software-In-The-Loop (SITL) simulator [58] was used in our experiments. The simulator uses the same autopilot used in the real drone to run a Copter. In the experiments, the QGroundControl [59] ground station, which is an open source ground control station (GCS) software application, was used.

We implement the search and rescue case study using the Ardupilot Software-In-The-Loop (SITL) [58] in which UAV flight path is secured and access to UAVs data is controlled through a smart contract. The cloud server is responsible for encrypting UAV data over Secure Socket Layer (SSL) and sending encrypted data to a distributed Off-chain database which is OrbitDB with IPFS. Indeed, we use IPFS to store UAV data while the hashes generated for stored data are kept on the blockchain network.

### 5.2. Blockchain System Setup

Hyperledger Fabric was applied because it has a modular design and it is permissioned which enables scalability and confidentiality. The fabric model consists of transactions, peers, assets, chaincode, ledger, channels, identity, membership, and consensus mechanisms. In essence, participants enroll via a trusted Membership Service Provider (MSP). The MSP component contains permissioned identities created by a trusted Certificate Authorities (CA) [60]. Indeed, Fabric CA is a private root CA provider responsible for creating digital identities (cryptographic material) that have the form of X.509 certificates for the fabric participants [61]. Besides, transactions could be in a private subnet of communication called channel [62]. Thanks to the concept of the channel, fabric enforces privacy and confidentiality.

Peers host copies of the ledger and copies of the chaincode. In the IoD environment, it is not suitable for each UAV to act as a peer as it will be difficult to control the blockchain network with many mobile nodes. That is why only the GCS and the cloud act as peers.

A peer might be an endorser or committer. In the first phase, endorsing peers receive, validate, and sign transactions. In the second phase, special nodes called orderers collect and order signed transactions into blocks and then send them to committing peers. In our case, GCS acts as an orderer node as it has a more secure connection to the network, enabling them to accept transactions more efficiently and to distribute blocks more easily.

In the third and final phase, committing peers receive the blocks and validate block conditions including double-spending and signature. Then the block is committed into the ledger [63].

Every peer has a copy of the ledger and a CouchDB state database. Furthermore, every peer includes smart contracts used to control access to the off-chain database and to write transactions to the blockchain ledger. Likewise, CouchDB presents actual data stored in a particular moment of time with rich query functionalities and also supports modeling of smart contracts as JavaScript Object Notation (JSON). In addition, the hyperledger framework presents a deployed blockchain business network that is manipulated by a set of tools called hyperledger composer [33]. The latter includes playground and composer-rest-server. In fact, the playground provides a graphic interface for the configuration, deployment, and testing of a blockchain business network. Likewise, composerrest-server is used to create from a Business Network Archive (BNA) file a REST API that is consumed by HTTP or REST Client. Thus, hyperledger composer-rest-server exposes blockchain services to the client application through REST API. In addition, Google fabric client using Remote Procedure Calls (gRPC) communicates with the fabric network [64]. Besides, the blockchain network generates notifications to the client via WebSocket [65].

In our system, smart contracts were designed and implemented via utilizing hyperledger composer. A smart contract is a self-executing computer program intended to perform a designed feature. It is defined within a chaincode and is compiled in the form of a BNA. Furthermore, a smart contract consists of four main parts including the model, the logic of transaction, the access control rules, and query definition. In fact, the model includes participants, assets, and transaction definition. The participants are the users that interact with the blockchain services. The assets are tangible or intangible things used between the participants and saved in blockchain registries. Transactions are logical operations defined in the smart contract. The fabric uses access control lists (ACLs) for resource access management that provides authentication and authorization to the participants and defines the roles of each participant within the business network. The fourth part of a smart contract is queries written in the bespoke query language in a separate file. The main responsibility of the queries is to select customized data based on user requests.

We propose to use OrbitDB with the Inter-Planetary File System (IPFS) as an Off-chain database. In fact, an off-chain database is used in order to store UAV’s collected data that is too large to be stored in the blockchain efficiently. OrbitDB is a distributed peer-to-peer database performing with IPFS. The latter generates the hash of the committed data into OrbitDB and stores it as an immutable transaction in the blockchain. When requesting to retrieve data from the off-chain database, access control policies will be executed by smart contracts (chaincode) to allow or deny data sharing. In IPFS, the uploaded files are fragmented. The maximum size of a fragment is 256 kilobytes. The fragment might contain only data, data, and links to other fragments, or only links. Every fragment is identified by a cryptographic hash also named content identifier. In order to reconstruct any file from its fragments, a Merkle directed acyclic graph (Merkle DAG) is used [66]. When a node divides the file into fragments and the Merkle DAG has been formed, the node is registered as a provider via the means of Distributed Hash Table (DHT). DHT consists of the information showing the connection between a node and a specific identifier. To ensure integrity, the Merkle DAG might be transmitted to the blockchain as a transaction.

**Figure 3:**
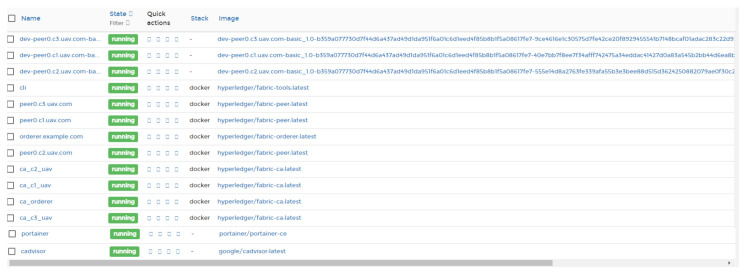
Blockchain Network Setup.

For our prototype, we built an experimental network consisting of three organizations namely c1.uav, c2.uav, and c3.uav and each organization having three peers each, each hosted in a docker container. We also have a Certificate Authority for each organization named ca_c1, ca_c2 and ca_c3. Our peers are named as peer 0, peer 1, and peer 2. The system configuration is illustrated in Figure 3.

## 6. Performance Evaluation

To validate the performance of our UTM-chain system, a number of experiments have been made. The designed model’s performance is evaluated using cAdvisor [67], which is an opensource benchmarking tool designed and developed to allow system users to measure the performance of blockchain-based application. The performance of UTM-chain over Hyperledger Fabric blockchain is analyzed based on resource utilization (memory utilization, CPU consumption, I.O., etc.), and latency.

The network latency can be defined as the total amount of time taken for a transaction to be executed in the Blockchain network. The network latency in the proposed system is categorized into two categories, i.e., transaction and read latency. The transaction latency is the total execution time across the Blockchain network that includes the transaction broadcasting time and consensus mechanism execution time, as presented in Equation (1).

Likewise, the read latency is defined as the addition of request submission time and time taken to get a response from the request, as computed in Equation (2).

(1) Transaction Latency=Network Threshold ∗ Transaction Confirmation Time − Transaction Submission Time

(2) Read Latency = Transaction Response Time − Transaction Submission Time

**Figure 4:**
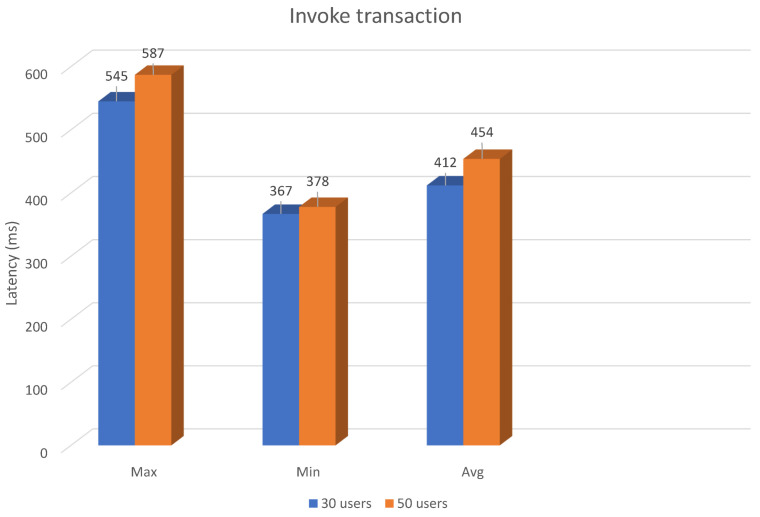
Latency in invoke transaction.

Three types of latency including minimum and maximum, and average latency of executing invoke transaction have been investigated and shown in Figure 4. Two groups including 30 and 50 users have been applied for the experiments. For the group of 30 users, the average latency is 412 ms while the average latency for the group of 50 users is 454 ms. The result shows that when the number of users rises, the average latency increases; nonetheless, the difference between two groups is small.

**Figure 5:**
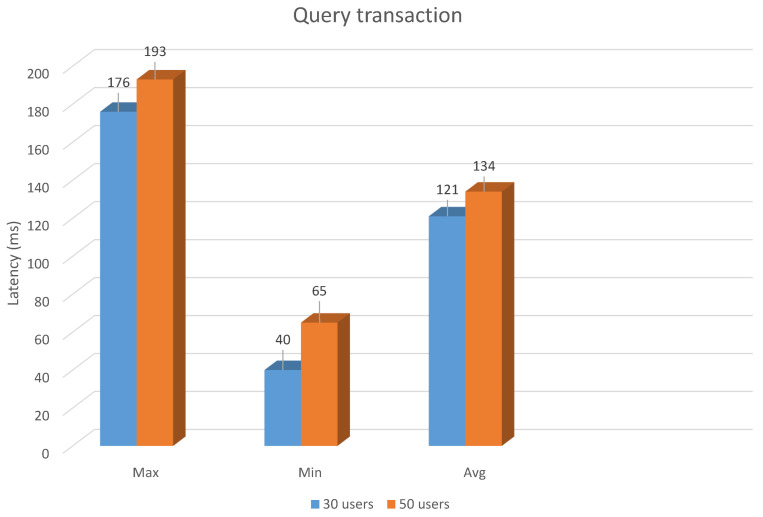
Latency in query transaction.

The latency of executing a query function for these groups is shown in Figure 5. The average latency of executing query transactions for two groups of 30 users and 50 users is 121 and 134, respectively. The minimum latency for the 30-user group and the 50-user group is 49 and 65 while the maximum latency for the 30-user group and the 50-user group is 176 and 193, respectively.

Additionally, we evaluate the performance of the proposed system in terms of resource utilization using cAdvisor. The experiments were performed in 10 iterations which includes the results related to memory consumption (avg, max) and central processing unit (CPU) (max, avg), as shown in Table 4. The average and maximum CPU consumption for peer is 7.32% and 11.34% respectively. Similarly, the maximum memory consumption (max, avg) for peer is recorded as 95.5 MB and 81.4 MB respectively. The ordering node’s average CPU utilization and memory consumption were 7.53% and 75.2 MB, while the CA node’s average CPU utilization and memory usage were 0% and 0 MB, respectively. The results demonstrate that the blockchain network has a reducing rate of resource consumption, higher reliability, and a promising user experience.

## 7. Security Analysis

This section represents the security analysis against the aforementioned attacks in the following subsection.

### 7.1. Protection against Availability Attacks

In availability, the information must be available when requested. Attacks on IoD against availability are Jamming and Denial of Service (DoS). Jamming of command signals from the Ground Control Station (GCS) or from other UAVs can affect the system availability. Blockchain does not prevent this attack but it minimizes threats to them. Considering the decentralization feature of blockchain, each UAV has a copy of the BC containing flight path details; thus, they can continue on its path in case of communication jamming.

In the case of DoS attack, the attacker overloads the UAV with unnecessary requests, making it unable to respond to legitimate GCS requests. As all participating members have the information of distributed BC, it is obvious to find that the requested information comes from the desired UAV in the network or from others.

### 7.2. Protecting Flight Data Integrity

In the post-flight phase, when the operator analyses flight data records, it needs to be sure that these data have not been modified by a third party. The immutability characteristic of blockchain ensures a level of data integrity and prevents data modification. Drone flight data are stored securely and accurately because each flight log is linked to the previous log with cryptography, which creates a verified data source for authorities.

### 7.3. Mitigating Data Privacy Attacks

During the search mission, the drone continuously sends different types of data, such as data collected from the drone’s sensors ( current altitude, speed, map position, weather, wind speed, images) and flight plan data (route, a destination address, visited locations, and target location) and flight status information ( current mission status, energy). This data is distributed using BC, which means that participating UAVs, GCS, and users have a copy of the ledger. Therefore, sensitive data is shared with all nodes in the network, without guaranteeing data privacy.

To prevent unauthorized nodes from accessing legitimate sensitive data in blockchain networks, which is also known as confidentiality, we use Hyperledger Fabric to preserve the privacy of UAV data. The key idea is that Hyperledger uses identity management and access control lists via private channels, allowing nodes to control and limit the access to their distributed sensitive information within the blockchain network. Through this approach, peers in the network know each other by their public identities, but they do not need to know the information disseminated over the network.

In addition, we investigate the use of an off-chain database to store sensitive UAV data in order to preserve the privacy of UAV data and to mitigate the storage burden on UAVs. This method is suitable to manage massive sensitive UAV data as they should have access restrictions because saving them inside the blockchain is not an efficient solution. Peers in the IoD can validate UAV data without the need of a central authority for approval, only by checking the blockchain, and UAV data is stored securely in the off-chain database.

## 8. Conclusions

In this work, we have proposed UTM-Chain, a blockchain-based security solution for an unmanned traffic management system. Blockchain technology has been integrated with UTM components to build an efficient and secure traffic monitoring system. This work also provides a road map towards efficient and secure management of UAV flight data records. The proposed architecture is based on a permissioned blockchain network. In the proposed architecture, blockchain is not used only for storing transactions but also it is used to control access to the flight data stored in a decentralized off-chain database through the execution of smart contracts. A decentralized off-chain database (OrbitDB with IPFS) and decentralized blockchain network improve the security level of our proposed architecture as centralized security issues are mitigated. In addition, several experiments have been done in order to evaluate the UTM-Chain framework performance via applying cAdvisor in terms of latency and resource utilization. The result shows that harnessing blockchain technology can help to improve the performance of the presented platform. Security analysis shows that the UTM-Chain solution provides a safe, efficient, reliable and tamper-resistant data security practice for unmanned traffic management systems and it can be a potential candidate for other IoD scenarios. 

## Figures and Tables

**Table 2:** Threats and vulnerabilities associated with the Unmanned Traffic Management system (UTM).

Target Components	Considered Threats and Attacks	Categories		Affected Security Parameter				
		Physical Attack	Cyber Attack	C	I	A	Auth	Privacy
**UAV and its sensors**	Dynamic obstacles Civic challenges Weather conditions Interference Battery depletion Physical access to the drone	✓				✓		
	GPS spoofing		✓	✓	✓		✓	
	GPS jamming		✓			✓		
	Hacking		✓	✓				
**GCS**	Software-related threats Virus, malware trojan, keylogger		✓	✓	✓	✓	✓	
**Communication** **Link**	Denial of Service (DoS/ DDoS)		✓			✓		
	Traffic analysis		✓	✓				✓
	Jamming		✓			✓		
	GCS Control Signals spoofing		✓	✓	✓		✓	
	Man in the middle		✓	✓	✓			
	Eavesdropping		✓	✓			✓	✓
	Hijacking		✓	✓		✓		
	Identity spoofing		✓	✓			✓	
	False location update		✓	✓				
**Cyber systems** **(cloud and internet)**	SQL injection		✓	✓	✓	✓		
	NoSQL injection		✓	✓	✓	✓		
	Insecure APIs		✓	✓	✓		✓	
	Malware Injection		✓	✓	✓	✓		

**Table 3:** Development Environment.

Tools	Description
Hyperledger Fabric	v1.2
Docker Engine	19.03.13
Docker Composer	1.27.4
CLI	bnc-hlf
Operating System	Ubuntu Linux 14.04 TLS
Simulator	SITL and QGroundControl

**Table 4:** Resource utilization analysis of the proposed system.

Type	Name	CPU (Max)	CPU (Avg)	Memory (Max)	Memory (Avg)
Docker	Peer0.c1.uav2.com	10.54%	7.32%	95.5 MB	81.4 MB
Docker	Peer0.c1.uav2.com	11.34%	5.76%	87.6 MB	77.3 MB
Docker	Peer0.c1.uav2.com	9.52%	6.34%	91.5 MB	65.8 MB
Docker	Peer0.c1.uav2.com_chaincode	9.23%	4.90%	57.3 MB	42.6 MB
Docker	Peer0.c1.uav2.com_chaincode	8.22%	3.23%	54.2 MB	41.5 MB
Docker	Peer0.c1.uav2.com_ chaincode	8.56%	5.22%	50.1 MB	42.2 MB
Docker	Orderer.example.com	15.32%	7.53%	92.7 MB	75.2 MB
Docker	ca_orderer	6.42%	0%	90.5 MB	0 MB
Docker	Ca_c1_uav	5.92%	0%	93.2 MB	0 MB
Docker	Ca_c2_uav	7.64%	0%	91.6 MB	0 MB
Docker	Ca_c3_uav	8.52%	0%	89.5 MB	0 MB

## Data Availability

Not applicable.
